# The impact of HBV infection on clinical outcomes of COVID‐19 patients: a systematic review and meta-analysis

**DOI:** 10.1017/S0950268823000705

**Published:** 2023-06-29

**Authors:** Yifan Guo, Xueling Zeng, Li Li, Linghang Wang

**Affiliations:** Emergency Department of Infectious Diseases of Beijing Ditan Hospital, Capital Medical University, Beijing, China

**Keywords:** COVID-19, hepatitis B virus, meta-analysis, systematic review

## Abstract

The impact of hepatitis B virus (HBV) infection on clinical outcomes of coronavirus disease 2019 (COVID-19) remains unclear. The aim of this study is to explore this impact. For this systematic review and meta-analysis, we searched PubMed, Web of Science, Embase, Cochrane library, China National Knowledge Infrastructure (CKNI), China Science and Technology Journal Database (VIP), and Wan Fang database for articles between 1 January 2020 and 1 February 2023. We used the Newcastle–Ottawa Quality Assessment to evaluate the study’s quality. A random-effects meta-analysis was performed utilising the rates of severe/critical illness and death in COVID-19 patients with and without HBV infection. Eighteen studies with a total of 40,502 participants met the inclusion criteria. The meta-analysis showed that compared to those without HBV infection, COVID-19 patients with HBV were at increased risk of mortality (OR = 1.65, I^2^ = 58%, and 95% CI 1.08–2.53) and severity (OR = 1.90, I^2^ = 44%, and 95% CI 1.62–2.24). The region and gender may influence the outcomes of COVID-19 patients with HBV infection, but it requires more global data to confirm. In conclusion, HBV infection is significantly linked to an increased risk of severity and mortality in COVID-19.

## Introduction

COVID-19 is an acute respiratory illness caused by the severe acute respiratory syndrome coronavirus 2 (SARS-CoV-2). A sizable fraction of COVID-19 patients experience liver damage, particularly those who have severe illness [1]. The possible pathophysiology of novel coronavirus-induced liver injury may be attributed to the expression of the angiotensin-converting enzyme 2 (ACE2) receptor in both hepatocytes and cholangiocytes, which could bind to SARS-CoV-2 [2].

Previous studies have revealed that, in addition to age, sex, and smoking history of patients, a number of pre-existing chronic diseases and conditions, such as cancer, asthma, obesity, kidney diseases, as well as hepatitis B virus (HBV) infection, may also be related to the severity and detrimental outcomes of COVID-19 [3]. HBV infection is of great concern in public health globally, causing more than one million deaths each year due to liver cancer and cirrhosis. Epidemiological studies on SARS patients indirectly showed that chronic HBV infection is a significant independent risk factor for the development to acute respiratory distress syndrome (ARDS) [4]. However, there are conflicting findings regarding the relationship between HBV infection and clinical outcomes of COVID-19 [5]. Therefore, it is critical to evaluate the effect of HBV infection on clinical outcomes of COVID-19 patients so that we could provide more preventive strategies and public health policy support to these patients. This meta-analysis aimed at investigating the relationship between clinical outcomes of COVID-19 patients and HBV infection.

## Methods

### Search strategy and study selection

Preferred Reporting Items for Systematic Reviews and Meta-Analyses (PRISMA) guidelines were used to conduct the systematic review [6]. The protocol has been registered in PROSPERO (CRD42022364786).

To increase the collection of valuable information globally, we searched PubMed, Web of Science, Embase, Cochrane library, CKNI, VIP, and Wan Fang database from 1 January 2020 to 1 February 2023, without limiting the language of the literature. The detailed search strategy is presented in the Supplementary material.

All the articles found through the electronic searches were exported to the Medical Literature King software. Search results were included if they met the following criteria: (1) Patients were diagnosed with COVID-19; (2) COVID-19 patients with or without HBV infection were divided into two groups in the study; (3) The outcome of hospitalised patients with COVID-19 were presented, including severe/critical illness and death. The way researchers define COVID-19 severity may differ, so we selected those articles which explicitly reported the number of patients among ‘severe /critical’ and those articles including ICU admission and mechanical ventilation patients. The following were the exclusion criteria of our study: (1) Studies that only included children, pregnant women, or other special groups; (2) Abstracts, guidelines, reviews, and case reports. Two researchers screened the literature individually, and discrepancies were settled by consensus with a third researcher.

### Data extraction and quality assessment

Two researchers independently extracted data and assessed the quality of the literature. Each included study provided the following information: (1) Basic study information, including first author, country, year of publication, study design, and subjects. (2) Events involving severe/critical patients, ICU admission, mechanical ventilation, and death.

Two researchers evaluated the quality of the studies using the Newcastle–Ottawa Scale (NOS). The NOS is a study scoring system that takes into account how well a researcher has accomplished their objectives in the areas of patient selection, comparability, and outcome [7]. The overall score was calculated, with a range of 0 to 3 representing high risk of bias, 7–9 representing low risk of bias, and 4–6 representing a medium risk of bias. Any disagreement was resolved by another researcher.

### Statistical analysis of data

The final extraction outcome data were imported into R‐software 4.2.2. The Cochrane’s χ^2^ test and I^2^ statistics were used to assess the heterogeneity of the studies. I^2^ value <50% indicates mild heterogeneity, while an I^2^ value ≥75% suggests significant heterogeneity. Moderate heterogeneity is considered if 50% ≤ I^2^ < 75%. The summary effect sizes and the odds ratio (OR) with 95% confidence interval (95%CI) of the clinical outcomes were estimated with random-effects models. A *P* value of less than 0.05 was regarded as statistically significant. Additionally, sensitivity analysis was carried out to evaluate the outcome’s stability by eliminating one study at a time. Egger’s test and funnel plot were applied to evaluate the potential publication bias.

## Results

**Figure 1.**
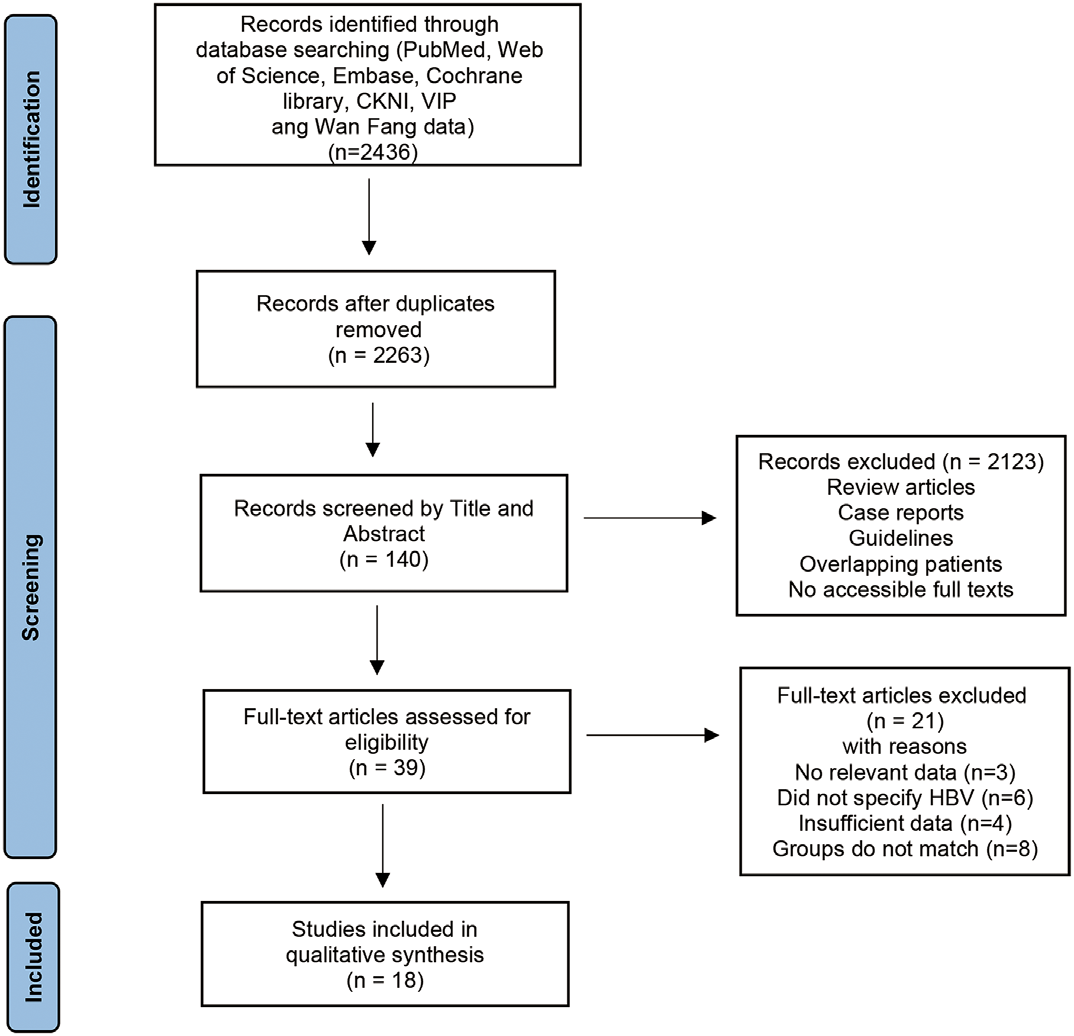
Flow chart for selection of studies. 157 × 157 mm (300 × 300 DPI).

On the first step, the systematic search identified a total of 2,436 results. After removing 173 duplicate studies, the remaining 2,263 articles were screened further by title and abstract, and 2,123 articles were excluded. Subsequently, according to full text content, 39 articles ineligible for this study were excluded. Finally, 18 studies that met the inclusion criteria were included. Figure 1 shows the complete study selection.

This meta-analysis included 40,502 patients, with sample sizes ranging from 28 to 19,160 [5, 8–24]. These studies were mainly from 3 countries – China, Korea, and Turkey. Details of the included studies are shown in Tables 1 and 2. The studies were all of high calibre, ranging from 7 to 9. Sensitivity analyses revealed that the overall estimations were unaffected significantly by removing one study at a time.

**Table 1.** Characteristics and demographic data of the included studies

Author	Country	Year	Sample size	Study design	NOS
Liping Chen	China	2020	326	Retrospective	7
Jung Wan Choe	South Korea	2022	19,160	Retrospective	7
Xiaoping Chen	China	2020	123	Retrospective	7
Muhammed Bekçibaşı	Turkey	2021	156	Retrospective	8
Yong Lin	China	2021	133	Retrospective	7
Qing He	China	2021	571	Retrospective	6
Jian Wu	China	2021	620	Retrospective	7
Jiaye Liu	China	2020	71	Retrospective	9
Yang Li	China	2020	28	Retrospective	7
Rui Liu	China	2020	106	Retrospective	7
Jing Wang	China	2022	436	Retrospective	7
Shanshan Yang	China	2022	2,899	Retrospective	9
Terry Cheuk-Fung Yip	China	2021	5,639	Retrospective	8
Seong Hee Kang	South Korea	2021	7,723	Retrospective	9
Rentao Yu	China	2021	67	Retrospective	9
Zeyang Ding	China	2021	2,073	Retrospective	9
Dong Ji	China	2020	140	Retrospective	8
Gupse Adali	Turkey	2021	231	Retrospective	7

NOS, Newcastle–Ottawa scale.

**Table 2.** Clinical outcomes of the included studies

Author	Male (*n*) (%)	HBV			non-HBV		
		Death	Severe /critical	ICU admission	Death	Severe /critical	ICU admission
Liping Chen	168 (51.53)	0	2	–	3	24	–
Jung Wan Choe	9,065 (47.31)	91	139	85	1,524	2,315	1,398
Xiaoping Chen	50 (40.65)	2	7	–	3	26	–
Muhammed Bekçibaşı	73 (46.79)	0	1	–	13	33	–
Yong Lin	72 (54.14)	0	0	–	0	0	–
Qing He	–	–	0	–	–	36	–
Jian Wu	315 (50.81)	0	23	–	14	84	–
Jiaye Liu	44 (61.97)	0	6	2	0	16	9
Yang Li	18 (64.29)	0	0	0	–	2	2
Rui Liu	60 (56.60)	4	23	–	4	27	–
Jing Wang	258 (59.17)	13	30	–	8	42	–
Shanshan Yang	1,478 (50.98)	29	246	56	37	541	70
Terry Cheuk-Fung Yip	2,743 (48.64)	29	–	–	109	–	–
Seong Hee Kang	–	12	26	–	225	454	–
Rentao Yu	35 (52.24)	0	4	–	0	25	–
Zeyang Ding	1,024 (49.40)	8	–	–	192	–	–
Dong Ji	82 (58.57)	0	–	–	1	–	–
Gupse Adali	153 (66.23)	6	–	–	15	–	–

HBV, hepatitis B virus; ICU, intensive care unit.

### Pre-existing HBV and mortality

**Figure 2.**
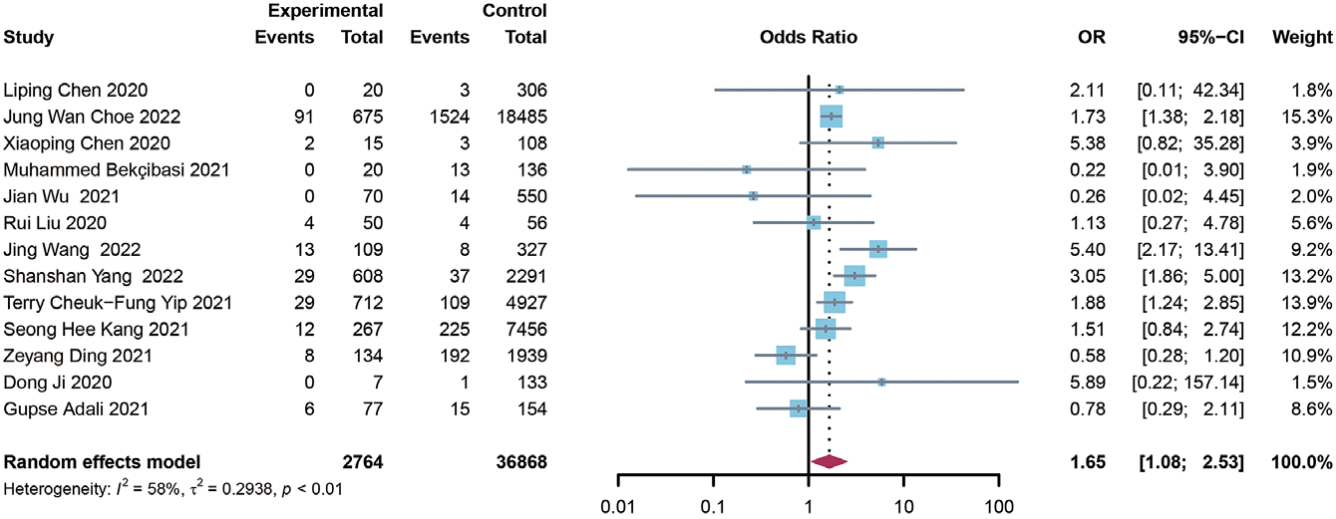
Forest plot showing the mortality of COVID-19 people with HBV infection. 265 × 101 mm (600 × 600 DPI).

**Figure 3.**
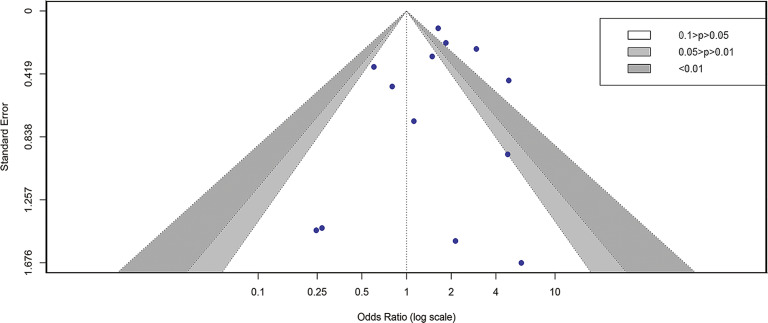
Funnel plot showing the mortality of COVID-19 people with HBV infection. 250 × 150 mm (300 × 300 DPI).

The aggregate analysis data of mortality included 13 studies [5, 8–10, 13–15, 17, 19, 20, 22–24]. In general, pre-existing HBV infection died at a significantly higher rate in patients with COVID-19 compared to those without HBV infection (OR = 1.65, I^2^= 58%, and 95% CI 1.08–2.53) (Figure 2). Egger’s test (P = 0.604) and the funnel plot revealed no publication bias in this analysis (Figure 3). Sensitivity analysis revealed that no particular study had an effect on the results (Supplementary Figure S1).

### Pre-existing HBV and the severity of COVID-19

**Figure 4.**
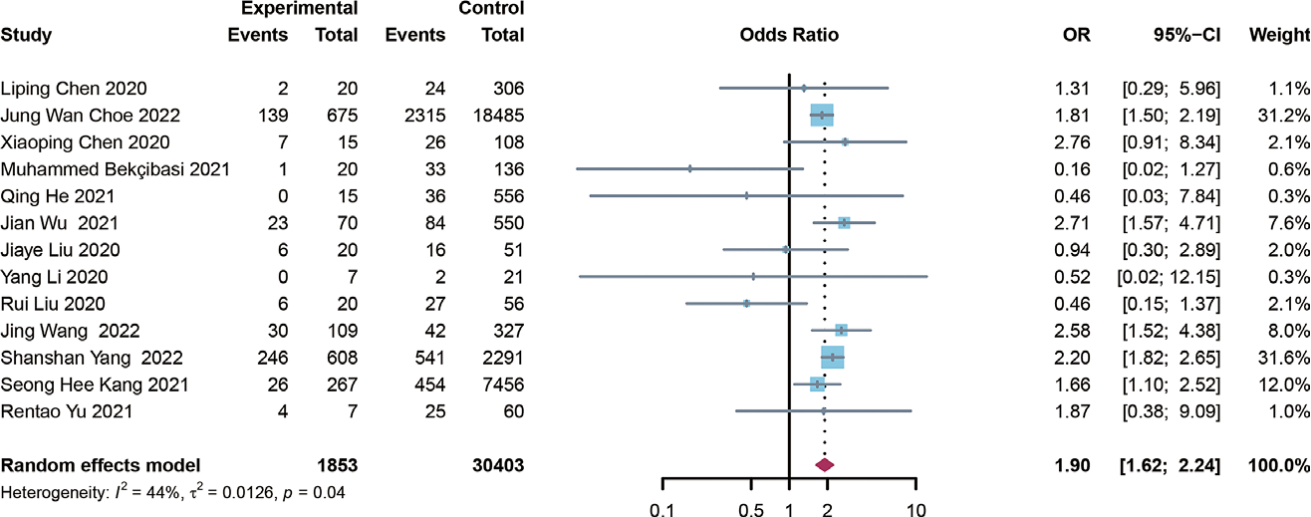
Forest plot showing the severity of COVID-19 people with HBV infection. 258 × 101 mm (600 × 600 DPI).

**Figure 5.**
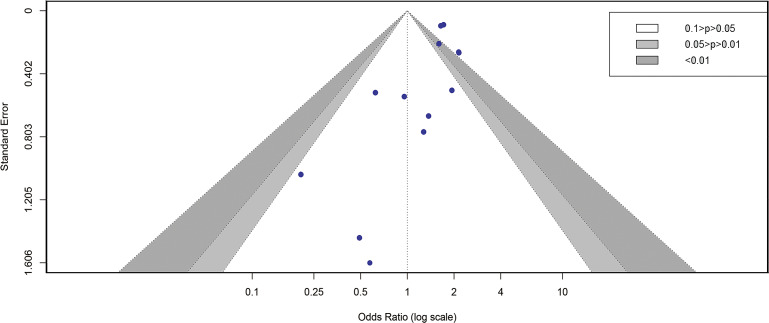
Funnel plot showing the severity of COVID-19 people with HBV infection. 250 × 149 mm (600 × 600 DPI).

13 studies were included in the analysis [8, 9, 11, 12, 14, 16, 17, 19–24] to determine the impact of pre-existing HBV on the severity of COVID-19 patients. Our study found that pre-existing HBV greatly enhances the risk of developing to severe COVID-19 (OR = 1.90, I^2^ = 44%, and 95% CI 1.62–2.24) (Figure 4). Egger’s test (P = 0.057) and the funnel plot indicated no publication bias in this study (Figure 5). The sensitivity analysis was performed by excluding each study at a time, showing that the outcome of the study was stable Supplementary Figure S2).

### Subgroup analysis

#### Region on clinical outcomes

Subgroup analysis indicated that Chinese COVID-19 patients with HBV showed higher mortality (OR = 1.95, 95% CI 1.03–3.70 versus OR = 1.55, 95% CI 1.17–2.07) and severity (OR = 2.13, 95% CI 1.81–2.51 versus OR = 1.75, 95% CI 1.48–2.09) than other areas (Supplementary Figure S3). However, the test for subgroup differences was not significant (χ^2^ = 0.41, P = 0.52 for mortality and χ^2^ = 2.61, P = 0.11 for severity). Due to the majority of studies on the impact of HBV on COVID-19, which have been conducted in Chinese patients, it is difficult to draw a conclusion about the influence of region.

#### Gender on clinical outcomes

We conducted a subgroup analysis to find out the impact of gender on clinical outcomes of COVID-19 patients with HBV. As shown in Supplementary Figure S4, gender was not associated with increased mortality in these patients (OR = 1.98, 95% CI 0.93–4.22 for male proportion ≥ 50% and OR = 1.38, 95% CI 0.73–2.60 for male proportion < 50%). However, the subgroup analysis showed that gender had an impact on severity (OR = 2.13, 95% CI 1.81–2.51 for male proportion ≥ 50% and OR = 1.24, 95% CI0.33–4.68 for male proportion < 50%). However, the test for subgroup differences failed to reach statistical significance (*P* = 0.36).

## Discussion

This meta-analysis included 18 studies between 1 January 2020 and 1 February 2023. According to the findings of our study, COVID-19 patients with HBV are at a higher risk of death and severe illness.

Up to 50% of COVID-19 patients showed liver enzyme abnormalities [25, 26]. A study showed that, even in mild cases, 23.5% of COVID-19 patients had abnormal liver enzymes, highlighting the fact that liver damage is not rare in COVID-19 patients [27]. Although both SARS-CoV-2 and HBV challenge liver physiology, current evidence is still inconclusive about the effect of HBV on clinical outcomes of COVID-19 patients. A report by Zhu JH, based on data from 6 epidemiological studies with 148 patients with COVID-19 and HBV infection, showed that HBV was not a significant risk factor for mortality or ICU admission among COVID-19 patients [28]. However, this study showed significant selection bias because it reported only in-hospital COVID-19 cases from China. Interestingly, another study by Yu et al. [29] with 37,696 COVID-19 patients of which 2,591 had HBV infection suggested that COVID-19 patients with HBV had a higher risk of developing severe disease. Our findings are consistent with the latter view and demonstrate that HBV infection may increase the adverse clinical outcomes of COVID-19 patients.

From an immunological aspect, during chronic HBV infection, T cells are unable to completely clear the virus and gradually develop into exhausted T cells, which lose effector function and memory T-cell characteristics before being deleted entirely. This process is known as T-cell exhaustion (TEX) [30]. Pathogen overstimulation is the primary cause of TEX, which is characterised by poor effector function, diminished proliferation and differentiation, decreased cytokine responses, and high expression of inhibitory receptors [31]. Such diminished immunity put HBV patients into a higher risk group for SARS-CoV-2-induced disease. Studies showed that the exhaustion of antigen-specific T cells caused by one pathogen or cancer could lead to exhaustion of other antigen-specific T cells through nonspecific pathways, such as the high-level co-expression of multiple inhibitory receptors like PD-1(CD279), cytotoxic T lymphocyte antigen-4 (CTLA-4, CD152), and lymphocyte-activation gene 3 (LAG3), which bring more severe repercussions [30, 32]. In addition, there is a risk of hepatitis B virus reactivation (HBVr) in severe COVID-19 patients when co-infected. HBVr is defined as an abrupt increase of virus replication in patients with stable HBV infection or previous HBV exposure, which can cause hepatic failure and death [33]. Some severe COVID-19 patients require immunosuppressive drugs to control cytokine overreacting response which may lead to HBV reactivation, increasing the risk of hepatitis deterioration, therefore worsening the clinical outcomes. A retrospective study showed, among 20 COVID-19 patients with chronic HBV, three patients developed HBV reactivation, and two of whom received methylprednisolone, which is the reason for the reactivation [12]. This is because of the host’s immune attenuation against HBV during immunosuppressive treatments, resulting in reactivation of HBV replication in hepatocytes and an increase in hepatocyte expression of hepatitis B virus antigens [34].

Furthermore, in order to assess the impact of region and gender on clinical outcomes of COVID-19 patients with HBV infection, subgroup studies were conducted depending on region and the proportion of males. Considering that HBV infection has a worldwide distribution, especially in China, we divided studies into China and other areas. Although meta‐analysis revealed an increased mortality (OR = 1.95 in China versus OR = 1.55 in other areas) and severity (OR = 2.13 in China versus OR = 1.75 in other areas) in the co‐infection for Chinese patients, the test for subgroup differences did not reach statistical significance, which requires more global data to confirm. We also discovered that male proportion has an impact on the severity of COVID-19 patients with HBV through a subgroup analysis with gender (OR = 1.98, 95% CI 0.93–4.22 for male proportion ≥ 50% and OR = 1.38, 95% CI 0.73–2.60 for male proportion < 50%). Previous studies have demonstrated significant gender differences in the clinical characteristics of Chinese patients with HBV-related liver diseases. Available global epidemiological data on SARS-CoV-2 show higher severity and mortality among males [25]. Indeed, females have quicker and stronger innate immune response than males, which may recognise viruses and release IFN and inflammatory cytokines more quickly, leading to a rapid virus clearance [35]. Moreover, studies showed that oestrogen protects against HBV infection progression by lowering HBV RNA transcription and inflammatory cytokine levels [36].

The objective of our study was to investigate the clinical outcomes in COVID-19 patients with HBV infection. The strength of our work was represented by the following aspects: (1) Inclusion of more epidemiological studies than previous meta-analyses on the same topic; (2) sensitivity analysis was carried out in each study, and the results of our study were stable after evaluation. The publication bias of the studies was also investigated to make the results more credible. (3) We also analysed the impact of region and gender on clinical outcomes of COVID-19 patients with HBV infection, to find out the focus of further research. However, there are a few limitations of our study that should be considered and discussed: First, all of the patients were Asian, so it is still unclear whether the results of this meta-analysis have global adaptability. Second, because most of the original studies did not provide sufficient information on HBV status, such as clinical phenotypes and treatment regimens, it is pity to draw additional conclusions about the effect of HBV infection on the course and prognosis of COVID-19. Third, SARS-CoV-2 has evolved continuously since the COVID-19 epidemic’s initial outbreak in December 2019, with many variants emerging all over the world. The characteristics of variants such as transmissibility, disease severity, and ability of immune evasion are different. Thus, it is plausible that the clinical outcomes may be different in COVID-19 patients with different variants. However, there are very few studies concerning this. Further research is still needed.

## Conclusion

HBV infection is associated with an increased risk of severity and mortality in COVID-19 patients. Extra attention should be paid to COVID-19 patients with HBV in order to improve their prognosis.

## Supporting information

Guo et al. supplementary material Guo et al. supplementary material

## Data Availability

Extracted data are available on request to the corresponding author.

## Supplementary material

For supplementary material accompanying this paper visit http://doi.org/10.1017/S0950268823000705. click here to view supplementary material
